# A commentary on the discrepancy between blood and tumour *BRCA* testing: An open question

**DOI:** 10.1111/1471-0528.17158

**Published:** 2022-04-05

**Authors:** Elisa De Paolis, Claudia Marchetti, Paola Concolino, Giovanni Scambia, Andrea Urbani, Anna Fagotti, Angelo Minucci

**Affiliations:** ^1^ Departmental Unit of Molecular and Genomic Diagnostics Fondazione Policlinico Universitario A. Gemelli IRCCS Rome Italy; ^2^ Division of Oncological Gynaecology Department of Women’s and Children’s Health Fondazione Policlinico Universitario A. Gemelli IRCCS Rome Italy; ^3^ Catholic University of the Sacred Heart Rome Italy

The molecular evaluation of breast cancer genes *BRCA1* and *BRCA2* represents a well‐known example of precision oncology. The availability of poly (ADP‐ribose) polymerase inhibitors (PARPi) as a targeted therapy option for several *BRCA*‐mutated cancer types (e.g. ovarian, breast, prostate and pancreatic cancer) has changed the course of *BRCA* testing over the few last years.^1^ In this context, an emerging path of molecular evaluation is represented by *BRCA* testing performed directly on tumour tissue (t*BRCA*): this increases the chance of identifying patients with a higher likelihood of benefiting from PARPi treatment. This approach leads to the simultaneous identification of both constitutional and somatically acquired variants, with a shorter turnaround time: the identification of *BRCA* pathogenic variants (PVs) could lead to secondary ‘reflex’ germline *BRCA* (g*BRCA*) testing in order to assess Personal and familial risks. In contrast, performing g*BRCA* as the first molecular test results in the loss of a relevant proportion of patients with tissue‐acquired *BRCA* PVs, in need of a follow‐up tumour test.^2^, ^3^, ^4^

In our opinion it is crucial to investigate the reliability of t*BRCA* in the identification of both somatic and germline variants. Inspired by Gourley’s recently published commentary,^5^ and taking into account that several troubling cases of discrepancy between blood and t*BRCA* testing have been reported, we have collected relevant recent studies covering the comparison between g*BRCA* and t*BRCA* to give a critical opinion about some shared key points of the somatic testing that could affect the final genotyping and reporting (Table 1).

**TABLE 1:** Comparative studies between germline and tumour *BRCA* testing results

Reference study	No. of subjects	Tumour type (sample type)	Germline test	Germline variants annotation	Tumour test	Somatic variant annotation	No. of missed germline variants	Reasons of discrepancy
Bekos et al.^2^	140	HGSOC (FFPE)	dHPLC+MLPA; Sanger+MLPA; NGS	ClinVar (ACMG)	NGS	BRCA Exchange; ClinVar; COSMIC; dbSNP (ACMG)	3	CNA detection; NGS data quality; input DNA quality
Fumagalli et al.^6^	62	EOC (FFPE)	N/A	N/A	NGS	ENIGMA; BRCA Exchange; ClinVar; LOVD (ACMG)	0	–
Peixoto et al.^7^	135	OC (FFPE)	Sanger+MLPA	N/A	NGS	N/A	0	–
Care et al.^8^	200	HGSOC (FFPE)	N/A	N/A (ACMG)	NGS+MLPA	ACMG	0	–
Rivera *et al* ^9^	67	EOC (FFPE)	NGS + MLPA	N/A	NGS	ENIGMA (ACMG)	0	–
Kim et al.^10^	57	POFTC (FFPE)	Sanger; NGS	BIC, ClinVar (ACMG)	NGS	BIC; ClinVar (ACMG)	1	Somatic reversion
Moore et al.^11^	341	POFTC (FFPE)	NGS	N/A	NGS	N/A	17	CNA detection; NGS data quality; input DNA quality; variant annotation
Eoh et al.^12^	98	HGSOC (FFPE)	Sanger	N/A	NGS	N/A	3	Somatic reversion
Frugtniet et al.^13^	169	HGSOC (FFPE)	NGS	ACMG	NGS	ACMG	1	CNA detection

*Note*: The table shows the recent relevant studies investigating concordance in the identification of *BRCA* germline variants between germline and tissue tests. For each reference study, the number of subjects with paired tumour and germline *BRCA* tests is reported, together with the cancer and specimen types. According to the study, we reported the methodological pipelines adopted, if available. The table also shows details about the germline findings not reported by tumour test.

Abbreviations: ACMG, American Collage of Medical Genetics and Genomics; dHPLC, denaturing high‐performance liquid chromatography; EOC, epithelial ovarian cancer; FFPE, formalin‐fixed paraffin‐embedded; HGSOC, high‐grade serous ovarian cancer; MLPA, multiplex ligation‐dependent probe amplification; NGS, next‐generation sequencing; OC, ovarian cancer; POFTC, epithelial peritoneal, ovarian and fallopian tube cancers.

Major reasons for discrepancy are related to: (i) differences in input DNA quality; (ii) characteristics of the next‐generation sequencing (NGS) approach; (iii) bioinformatics pipeline features (e.g. the ability to predict the occurrence of copy number alterations (CNAs) and the evaluation of the intron/exon boundaries); and, finally, (iv) issues related to the interpretation and classification of *BRCA* variants.

Currently, t*BRCA* testing is mainly performed on two sample types: fresh frozen tissue (FFT) and formalin‐fixed paraffin‐embedded (FFPE) tissue, with the focus here on FFPE, being the most common tissue type in clinical diagnostic use. As part of the pre‐analytic phase there are established guidelines with regards to tissue fixation steps, tissue section size and the assessment of neoplastic cell content. Suboptimal DNA quality, leading to inaccurate t*BRCA* analysis, causes around 5% of FFPE t*BRCA* NGS testing failures, with the consequent need for additional new samples.^9^ In the study performed by Bekos et al.,^2^ only the retesting of newly extracted tumour DNA resolved two cases of discrepancy with g*BRCA*. In the study performed by Care et al.,^8^ the test failure rate was related to the fixation methods or storage of FFPE material. Ad hoc recommendations for the ‘ideal’ starting tissue material are available.^9^, ^14^

Furthermore, the analytical steps of *BRCA* gene amplification and sequencing should be performed using different approaches, according to the methodological procedures of the laboratory. These may include several types of sequencing chemistry (e.g. amplicon‐based and capture‐based sequencing), platforms (e.g. Illumina and IonTorrent) and data analysis pipelines (e.g. full‐coding regions or hot‐spot analysis, different size of splice site region analysed and detection of copy‐number alterations). Each of the above methods has specific pitfalls that can affect the downstream bioinformatics filtering and calling of variants. For example, in amplicon‐based approaches, the failure to detect a variant may be related to the experimental design of the primer distribution along the genomic region of interest. Variants located at the 3′ or 5′ ends of overlapping amplicons could be covered by just one read and could consequently be identified with a ‘strand bias’ flag and filtered out at the bioinformatics quality check.^3^

The use of different bioinformatics pipelines for the NGS data analysis of germline and somatic tests in the same patient could be the cause of apparently inconsistent results, as emerged in the work of Lincoln et al.^15^ Moreover, in the case of discrepancy involving splice site variants, it could be useful to check the concordance of the splice site region size included in the germline and somatic bioinformatics pipelines.^3^ Regarding data analysis, it should be acknowledged that some tumour testing platforms filter out germline variants in the final reports in order to improve the accuracy of somatic variant calling.

Another well‐known cause of g*BRCA*/t*BRCA* non‐concordance arises from the bioinformatics‐assisted calling of CNAs in tissue samples.^2^, ^3^, ^15^

The sensitivity of NGS in detecting CNAs mostly depends on the quality of the DNA, tumour heterogeneity, low neoplastic cell content, library preparation, type of algorithm and size of rearrangement. As a consequence, the somatic bioinformatics pipeline requires the ad hoc development of computational algorithms for the specific characteristics of the raw sequencing data (e.g. maximum volume, coverage uniformity and sufficient read depth).^1^ Even if the majority of the methods are optimised for somatic CNA identification,^6^, ^8^ attention should be paid to the comparison of blood and tissue tests results.^13^ For example, in the study reported by Bekos et al.,^2^ a verified single exon pathogenic germline deletion of *BRCA1* was not identified in a tumour sample, but a careful re‐evaluation of the bioinformatics variant calls solved the discrepancy.

A relevant role in the evaluation of non‐concordant results is played by the post‐analytical steps used for the interpretation of *BRCA* variants. Complex issues underly the classification of *BRCA* variants. The American College of Medical Genetics and Genomics (ACMG) and the Association for Molecular Pathology (AMP) have established the best practice for germline variant interpretation, providing a well‐known classification using a five‐tier system.^16^ Conversely, the interpretation of somatic variants should be focused on their impact on clinical care. Specifically, the evidence‐based categorisation of somatic variants released by the AMP, the American Society of Clinical Oncology (ASCO) and the College of American Pathologists (CAP) includes a four‐tier system: (i) variants of strong clinical significance (levels A and B of evidence); (ii) variants of potential clinical significance (levels C and D of evidence); (iii) variants of unknown clinical significance; and (iv) variants that are or are likely to be benign.^17^ With the publication of an increasing number of large‐scale tumour sequencing projects, considerable information is being collected into publicly available databases that are useful for querying the significance of a *BRCA* variant. Cancer‐specific variant databases include BRCAexchange, OncoKB, Catalogue of Somatic Mutations in Cancer, My Cancer Genome, cBioPortal, Memorial Sloan Kettering Cancer Center, International Cancer Genome Consortium and VARSOME, whereas constitutional variant databases include ClinVar, Human Gene Mutation Database, ENIGMA, Leiden Open Variation Database, gnomAD, CanVig‐UK and VARSOME. Differences in germline‐ and somatic‐based annotation may exist between the above‐mentioned tools, which could increase the risk of non‐concordant annotation of a *BRCA* variant. This is crucial in t*BRCA* and g*BRCA* concordance evaluation, when the same molecular test is performed in different laboratories: variants that met the criteria to be considered oncogenic in the somatic test may not meet the strict germline criteria to be considered pathogenic. This situation could more likely affect the missense variants of unknown significance (VUSs).^15^ ^,^ ^16^ ^,^ ^17^ As reported by Bekos et al.,^2^ after the inclusion of *BRCA* VUSs in the secondary data analyses, the concordance rate of tumour testing compared with the germline decreased, mainly through VUS classification. In a large study investigating the differences in germline and somatic variant interpretation, Moody et al. highlighted a relevant percentage of discrepancies in variant classification.^15^

Additionally, Kim et al. reported a case of discrepancy derived from a true reversion of the germline *BRCA1* variant, found through the restoration of the wild‐type allele in the tissue cells.^10^ Several patients acquired PARPi resistance with prolonged oral administration of PARPi. In this context, somatic reversion of a germline variant represents a significant reason for discrepancy and the loss of PARPi sensitivity.

Finally, t*BRCA* reporting should follow specific criteria that maximise molecular information, improving the clinical relevance of the test and giving a more comprehensive interpretation of each variant. With these aims, a peculiar role is played by the ‘naturally occurring’ *BRCA* splicing isoforms: careful consideration should be given to rare variants that are characterised by variability in the final effect and annotation in the context of all gene‐relevant transcripts.^18^

In conclusion, the accurate detection and evaluation of g*BRCA* and t*BRCA* variants depends on multiple factors. We argue that only harmonised guidelines encompassing the above‐mentioned methodological and post‐analytical steps could optimise this process and help resolve the *BRCA* germline and somatic testing bias. In our laboratory, *BRCA* genetic testing is routinely performed on blood, FFT and FFPE samples.^1^ In many cases, we routinely analyse matched blood and tissue samples from the same patient, in order to perform an efficient *BRCA* test that comprehensive for both germline and somatic evaluation. This approach highlights the need for multidisciplinary and skilled resources to obtain a solid molecular characterisation of the tumour. Together with the need for standardization, we suggest performing t*BRCA* and *gBRCA* testing in the same laboratory to improve the reliability of the entire molecular path taken by patients and their clinicians.

## CONFLICT OF INTERESTS

None declared. Completed disclosure of interests form available to view online as supporting information.

## AUTHOR CONTRIBUTION

EDP and AM conceived and wrote the commentary with support from PC. CM, GS, AU and AF supervised the project. All authors discussed, edited and contributed to the final article.

## ETHICS APPROVAL

Not applicable.

## GLOSSARY

**Breast cancer susceptibility genes 1 and 2 (*BRCA1* and *BRCA2*)**, *BRCA1* and *BRCA2* are tumour suppressor genes implicated in several cellular processes, including transcription, protein ubiquitination, cell‐cycle regulation and DNA damage response, with a particularly relevant role in DNA repair during homologous recombination; **Germline pathogenic variants (gPVs) in *BRCA* genes**, Specific types of variants (e.g. non‐sense, frameshift, canonical ±1 or 2 splice sites, initiation codon and single exon or multi‐exon deletion) disrupt *BRCA* gene function by leading to a complete absence of the gene product through a lack of transcription or non‐sense‐mediated decay. These variants are inherited and increase the risk for breast, ovarian, pancreatic and prostate cancers; **Tissue pathogenic variants (tPVs) in *BRCA* genes**, Specific types of variants (e.g. non‐sense, frameshift, canonical ±1 or 2 splice sites, initiation codon and single exon or multi‐exon deletion) disrupt *BRCA* gene function by leading to a complete absence of the gene product through a lack of transcription or non‐sense‐mediated decay. These variants are unique to the tumour; **Variants of unknown significance (VUSs)**, VUSs are variants that we are still not able to clearly classify as pathogenic or non‐pathogenic because of poor experimental and clinical data. As a consequence, the impact of VUSs on an individual’s cancer risk is not yet known; **Copy number alterations (CNAs)**, CNAs are a subtype of unbalanced structural rearrangements of the genome, characterised by insertions or deletions of a large DNA segment. Currently, the size of CNA rearrangements range from 50 bp to several Mb; **Tumour *BRCA* (t*BRCA*) testing**, t*BRCA* testing is the molecular evaluation of *BRCA* genes from tumour tissue, mainly on formalin‐fixed paraffin‐embedded (FFPE) samples. This approach allows the identification of inherited and non‐inherited *BRCA* PVs; **Germline *BRCA* (g*BRCA*) testing**, g*BRCA* testing is the molecular evaluation of *BRCA* genes mainly from blood samples. This test allows the identification of the inherited *BRCA* variants alone (i.e. variants occurring in germ cells and passing from generation to generation), and rarely finds new variants; **Reflex g*BRCA* testing**, Reflex g*BRCA* testing is the mandatory confirmatory test performed on blood samples in order to assess the germline or somatic origin of the molecular alteration previously identified in the tumour; **Next‐generation sequencing (NGS)**, NGS is a high‐throughput DNA/RNA sequencing technology that allows the parallel analysis of large regions of the genome in multiple samples per run at significantly reduced cost and higher sensitivity compared with traditional methods (e.g. Sanger sequencing); **Poly (ADP‐ribose) polymerase inhibitors (PARPi)**, PARPi are a novel class of anti‐cancer drugs that compete with NAD+ for the catalytically active site of PARP molecules. PARPi have been approved by the US Food and Drug Administration (FDA) for use in patients with germline and/or somatic *BRCA*‐mutant ovarian, breast, pancreatic and prostate cancers in a synthetically lethal interaction

## Supporting information


**Appendix** S1

**Appendix** S2

**Appendix** S3

**Appendix** S4

**Appendix** S5

**Appendix** S6

**Appendix** S7

## Data Availability

Data sharing not applicable ‐ no new data generated.
